# Gastric Antral Vascular Ectasia Preceding the Diagnosis of Limited Cutaneous Systemic Sclerosis

**DOI:** 10.7759/cureus.96021

**Published:** 2025-11-03

**Authors:** Nudrat Khan, Arslan Ather, John Pradeep

**Affiliations:** 1 Acute Medicine, North West Anglia NHS Foundation Trust, Peterborough City Hospital, Peterborough, GBR; 2 Rheumatology, North West Anglia NHS Foundation Trust, Peterborough City Hospital, Peterborough, GBR

**Keywords:** argon plasma coagulation (apc), gastric antral vascular ectasia, iron-deficiency anaemia, oesophago-gastro-duodenoscopy (ogd), raynaud’s phenomenon, systemic sclerosis

## Abstract

Systemic sclerosis is an autoimmune connective tissue disease that affects multiple organ systems, leading to diverse clinical presentations. Raynaud’s phenomenon is one of the most common early manifestations, reflecting the underlying vasculopathy. In rare cases, vascular changes also involve internal organs, including the gastrointestinal tract. We present the case of a 79-year-old female who first presented in 2015 with recurrent iron-deficiency anaemia requiring multiple blood transfusions. Oesophago-gastro-duodenoscopy revealed features of “watermelon stomach,” consistent with gastric antral vascular ectasia (GAVE). She underwent multiple sessions of argon plasma coagulation to control bleeding. Seven to eight years later, she developed classical features of systemic sclerosis, including sclerodactyly, worsening Raynaud’s phenomenon, skin calcifications, and digital ulcerations. Autoimmune testing was positive for antinuclear antibodies, anti-Ro, and anti-centromere antibodies, confirming limited cutaneous systemic sclerosis. This case demonstrates the potential for GAVE to appear before other features of systemic sclerosis become clinically evident. Patients presenting with idiopathic or recurrent GAVE should be evaluated for underlying connective tissue disease, as timely recognition can influence monitoring, treatment, and long-term outcomes.

## Introduction

Systemic sclerosis is a multisystem autoimmune disease characterised by skin fibrosis and vasculopathy. Common presenting features include Raynaud’s phenomenon, sclerodactyly, other skin manifestations, and arthralgia. Less common but more severe manifestations include pulmonary hypertension, lung fibrosis, and gastrointestinal symptoms. The most common gastrointestinal manifestations are swallowing difficulty due to oesophageal dysmotility. However, rarely, it can present with manifestations of vasculopathy affecting gastrointestinal blood vessels. This can manifest as gastric antral vascular ectasia (GAVE), also known as “watermelon stomach,” a vascular abnormality of the gastric antrum that can lead to iron-deficiency anaemia due to chronic blood loss. Diagnosis is usually confirmed on oesophageal gastrointestinal duodenoscopy showing features of watermelon stomach or GAVE. The reported prevalence of GAVE among systemic sclerosis patients varies from 1% to 22.3% [1]. Despite this, GAVE remains an under-recognised and underappreciated manifestation of the disease and rarely presents before other classical features of systemic sclerosis.

## Case presentation

An 89-year-old female in 2015, at the age of 79 years, developed symptomatic anaemia with a haemoglobin drop below 80 g/L. She was found to have microcytic iron-deficiency anaemia with recurrent drops in her haemoglobin requiring blood transfusions. She had a past medical history of osteopenia, hypothyroidism, hypertension, ischaemic stroke, and vestibular schwannoma.

**Figure 1:**
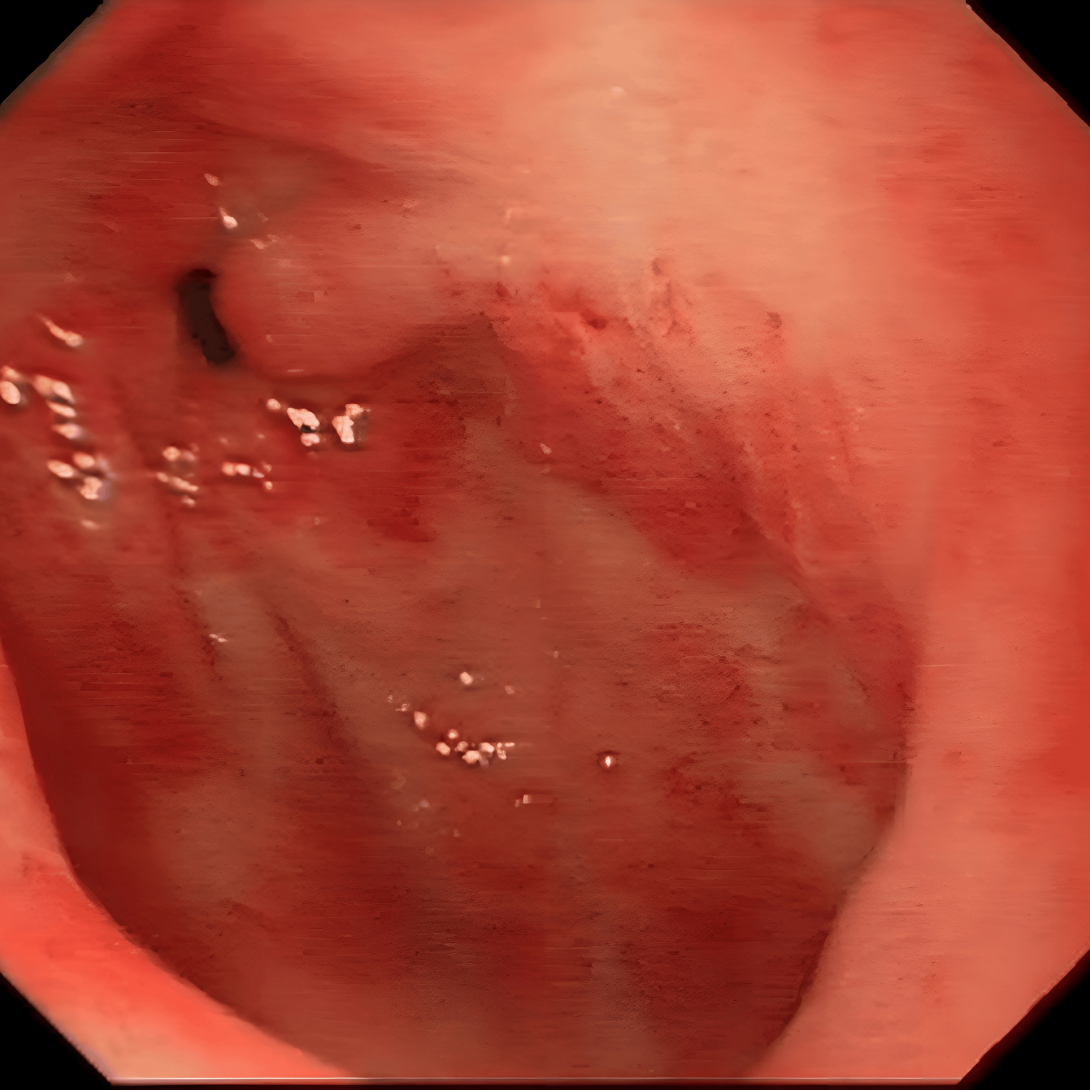
Gastric antral vascular ectasia lesion observed on upper gastrointestinal endoscopy (16.03.2019). Endoscopic image showing a classic gastric antral vascular ectasia lesion in the gastric antrum before argon plasma coagulation treatment.

**Figure 2:**
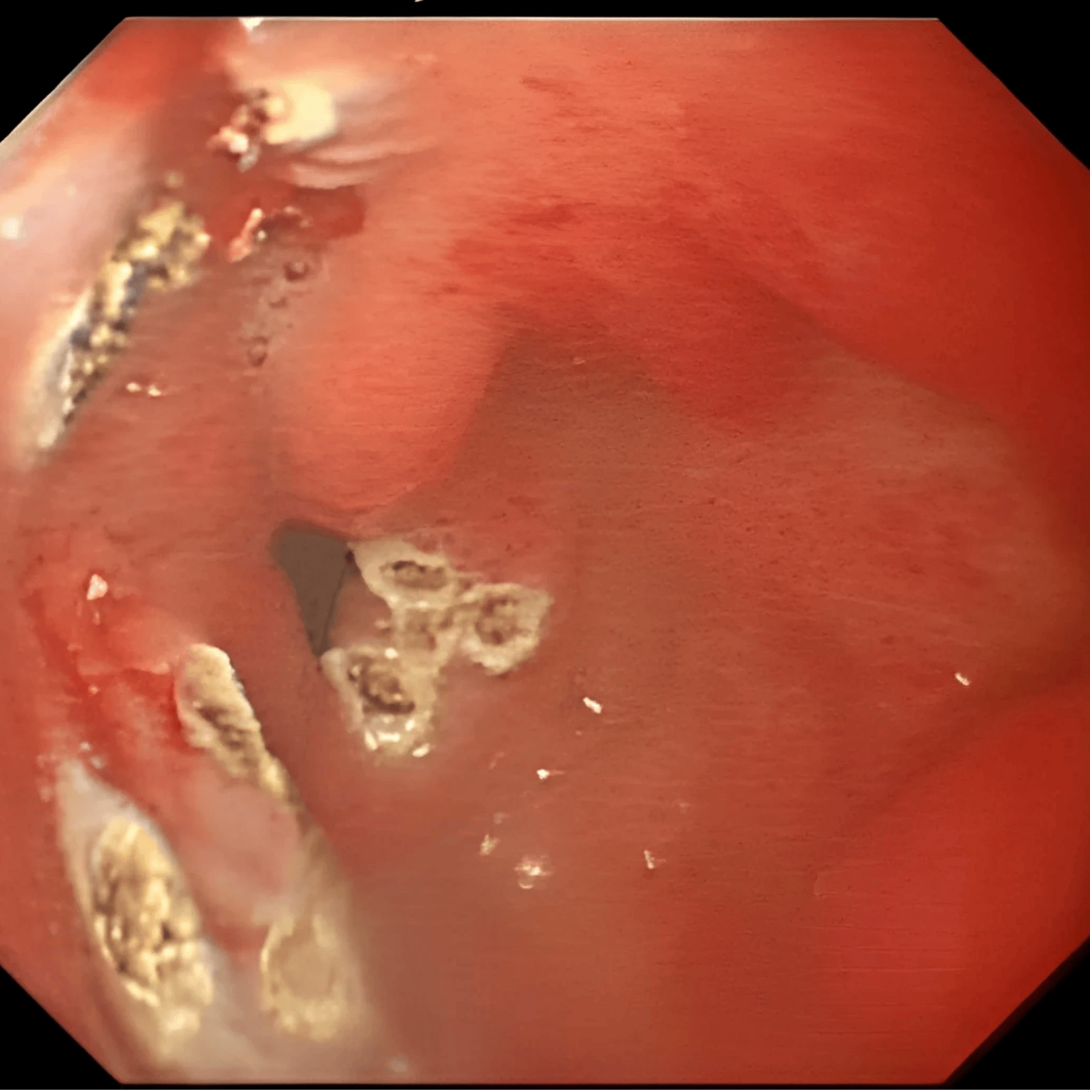
Evidence of scarring from previous banding for gastric antral vascular ectasia on upper gastrointestinal endoscopy. Endoscopic image showing fibrotic scarring in the gastric antrum from prior endoscopic intervention (documented as banding in the endoscopy report).

Her initial workup did not show any evidence of malignancy. However, oesophago-gastro-duodenoscopy (OGD) showed findings in keeping with GAVE (Figure 1), with evidence of fibrotic scarring from previous endoscopic banding (Figure 2). A capsule endoscopy in June 2015 was unremarkable, and her anaemia was attributed to GAVE. She required multiple sessions of argon plasma coagulation (APC). She also required multiple blood transfusions and courses of parenteral iron to treat her iron-deficiency anaemia.

Approximately eight years later, in 2023, she presented with worsening Raynaud’s phenomenon, associated with painful digital ulceration affecting the left middle finger and right hand. Over the preceding two years, she had noticed progressive skin tightening affecting both upper and lower limbs and painful lesions on both elbows. These new clinical features prompted further autoimmune workup.

There were no associated joint pains or oral ulcers, though she reported exertional breathlessness likely secondary to anaemia. There was no history of chest pain, cough, or haemoptysis. Examination confirmed skin tightening and thickening over the feet, extending up to the mid-shin, and a healed digital ulcer was noted on the left middle finger. She also had skin thickening extending to the mid-forearms along with calcinotic lesions on both elbows. There was no oral puckering. Cardiopulmonary examination was unremarkable.

Autoimmune serology was performed, which showed positivity for antinuclear antibody, anti-Ro antibody, and anti-centromere antibody, consistent with limited cutaneous systemic sclerosis. Parietal cell antibodies were negative. Haptoglobin, lactate dehydrogenase, and bilirubin were within normal limits. The myositis panel was negative. Further workup showed no evidence of interstitial lung disease on CT of the chest. Blood test again demonstrated stable iron-deficiency anaemia and chronic kidney disease stage 2. However, she had no significant proteinuria.

She was diagnosed with limited cutaneous systemic sclerosis with manifestations including sclerodactyly, skin calcinosis, Raynaud’s with digital ulcerations, and gastrointestinal involvement in the form of GAVE. She was offered symptomatic treatment with iloprost to improve Raynaud’s and digital ulcerations. However, she declined this due to concerns regarding side effects. Sildenafil was contraindicated due to the past medical history of ischaemic stroke. After discussion with Dermatology, a trial of clobetasol propionate cream once daily or fludroxycortide tape overnight for the ulcerated finger lesion was advised. As the skin tightening was limited and not significantly bothersome, she was not considered for any immunosuppression.

## Discussion

Vasculopathy is a key component of the pathophysiology of systemic sclerosis. Autoimmune-mediated vascular damage and endothelial dysfunction can affect blood vessels, causing vasculopathy [2]. Although the peripheral vasculature of the skin is most commonly involved, it can affect blood vessels in any organ. Consequently, its presentation is highly variable depending on the site of vascular involvement. GAVE represents one of the under-recognised manifestations of this vasculopathy when it involves the gastrointestinal system. This can cause recurrent and severe iron-deficiency anaemia due to chronic blood loss. This sometimes requires blood transfusion along with iron supplementation [3].

Diagnosis is usually established on OGD, which typically shows the characteristic “watermelon stomach” appearance [4]. Along with the conservative management with blood transfusion and iron supplements, it requires APC to stop the bleeding from blood vessels [5]. Different studies have suggested variable prevalence of GAVE in patients with systemic sclerosis, ranging from 1% to 22.3% [1]. The underlying pathogenesis of GAVE in systemic sclerosis remains incompletely understood. One possible hypothesis is that early microvascular injury and endothelial activation in the gastric mucosa may precede the development of systemic or cutaneous features, which can explain why GAVE can occasionally present as an initial or isolated manifestation of systemic sclerosis.

In our patient, although Raynaud’s phenomenon had been present since early adulthood, it had remained mild, stable, and uninvestigated for decades, consistent with primary Raynaud’s. GAVE developed at the age of 79 years and required multiple interventions for symptomatic anaemia. It was only approximately eight years later that progressive skin tightening, digital ulceration, and positive antibodies emerged, confirming a diagnosis of limited cutaneous systemic sclerosis. This sequence illustrates that GAVE can be the first clinically recognisable manifestation of systemic sclerosis, even when subtle vascular symptoms, such as primary Raynaud’s, have been present for decades.

This case highlights the importance of considering systemic sclerosis in patients presenting with idiopathic or recurrent GAVE, particularly in the presence of any subtle vascular or autoimmune features [2]. Early recognition of gastrointestinal vasculopathy may allow for closer monitoring, timely diagnosis, and appropriate management of evolving systemic disease. Clinicians should be aware that the absence of classical cutaneous features at initial presentation does not exclude systemic sclerosis and that vascular manifestations may precede skin involvement in some cases [6].

Conversely, young patients presenting with Raynaud’s phenomenon should be assessed for secondary causes, particularly when features are severe, asymmetric, or accompanied by other vascular or autoimmune signs [7]. Early evaluation may facilitate the timely identification of evolving systemic sclerosis before the onset of classical cutaneous manifestations.

## Conclusions

GAVE is an under-recognised manifestation of systemic sclerosis and may occasionally precede the development or recognition of other classical features. Patients presenting with idiopathic or recurrent GAVE should be evaluated for underlying connective tissue disease, as timely recognition can influence monitoring, treatment, and long-term outcomes. Conversely, individuals with long-standing Raynaud’s phenomenon should also be assessed for potential secondary causes, as evolving systemic sclerosis may remain subclinical for several years.
